# Late metabolic precursors for selective aromatic residue labeling

**DOI:** 10.1007/s10858-018-0188-z

**Published:** 2018-05-28

**Authors:** Julia Schörghuber, Leonhard Geist, Gerald Platzer, Michael Feichtinger, Marilena Bisaccia, Lukas Scheibelberger, Frederik Weber, Robert Konrat, Roman J. Lichtenecker

**Affiliations:** 1 Institute of Organic Chemistry, University of Vienna, Währinger Str. 38, 1090 Vienna, Austria; 2 Christian Doppler Laboratory for High-Content Structural Biology and Biotechnology, Department of Structural and Computational Biology, Max F. Perutz Laboratories, University of Vienna, Dr-Bohr-Gasse 9, 1030 Vienna, Austria

**Keywords:** Protein labeling, Aromatic residues, Protein overexpression, Chemical shift mapping, Ligand induced cross-correlation, Intrinsically disordered proteins

## Abstract

In recent years, we developed a toolbox of heavy isotope containing compounds, which serve as metabolic amino acid precursors in the *E. coli*-based overexpression of aromatic residue labeled proteins. Our labeling techniques show excellent results both in terms of selectivity and isotope incorporation levels. They are additionally distinguished by low sample production costs and meet the economic demands to further implement protein NMR spectroscopy as a routinely used method in drug development processes. Different isotopologues allow for the assembly of optimized protein samples, which fulfill the requirements of various NMR experiments to elucidate protein structures, analyze conformational dynamics, or probe interaction surfaces. In the present article, we want to summarize the precursors we developed so far and give examples of their special value in the probing of protein–ligand interaction.

## Introduction

The structure and interplay of proteins determine the cell’s proliferation, development, function and fate. A deep understanding of their complex conformational properties and interaction networks represents the key issue to unravel the principles of life at a molecular level. Only if the properties of the single nodes in e.g. protein signal cascades or metabolic networks are known and the interaction mechanisms understood, subtle changes leading to pathogenic progression and disease development can be addressed by developing non-endogenous therapeutic compounds. NMR spectroscopy is one of the three main methods, next to X-ray diffraction and cryo-electron microscopy, to investigate the properties of proteins at an atomic, or near-atomic resolution (Banci et al. 2010). The special value of protein NMR is given by the highly diverse set of possible pulse sequences, which can give information about structural properties, dynamic processes and the interaction with binding partners. This information can be obtained under near native conditions from samples in aqueous buffer solution. However, the NMR-based elucidation of proteins is limited by sensitivity and resolution issues (Ardenkjaer-Larsen et al. 2015), which are partly compensated by constant improvement of experimental techniques, hardware development and sample preparation (Campbell 2013), especially concerning novel developments in protein isotope labeling techniques (Ohki and Kainosho 2008).

Introducing ^13^C, ^15^N and ^2^H at defined atomic positions improves signal resolution and leads to spectra simplification. As a result, NMR signals are attributed to the corresponding nuclei more easily and can be transferred to automated assignment algorithms (Güntert 2009). Highly selective labeling shifts the molecular weight limit of proteins which are amenable to structure calculation based on NMR data. The number of high quality structure restraints can be increased in this case resulting in more accurate protein structures. Furthermore, most of the diverse NMR experiments, which have been developed to probe protein dynamics at different time scales require isolated spin systems (Ishima et al. 2001). The analysis of these ^13^C–^1^H or ^15^N–^1^H spin pairs is greatly simplified due to the lack of one-bond ^13^C–^13^C or three-bond ^13^C–^1^H coupling, and thus allows for an accurate interpretation of the corresponding relaxation dispersion rates. The importance of protein NMR in the drug development process is constantly increasing as this method provides valuable information about binding sites and large interaction surfaces (Pellecchia et al. 2002). However, protein NMR is associated with high costs and still far from being a high throughput method. Highly selective, economic protein labeling can improve the situation by decreasing the minimal sample concentrations required for certain NMR experiments.

Two main complementary methods have been developed to implement defined protein isotope patterns. In cell-based approaches, a host organism is grown in media containing suitable isotopologues of metabolic amino acid precursors. After cellular uptake, these compounds are converted into the target residues within their metabolic pathways in-vivo. Such overexpression systems have been described for prokaryotic (*E. coli*) (Hoogstraten and Johnson 2008; Mondal et al. 2013), as well as eukaryotic cells (yeast, insect cell-lines) (Morgan et al. 2000; Takahashi and Shimada 2010). Especially when early metabolic intermediates are used as labeled nutrients, the danger of cross-labeling to unwanted positions is very high, thus resulting in unselective isotope patterns. Cross-labeling is avoided in the second method, which uses cell lysates in-vitro to generate the target proteins from isotope containing amino acids (Kainosho et al. 2006; Kainosho and Güntert 2009; Staunton et al. 2006; Takeda et al. 2010; Torizawa et al. 2004, 2005). These cell-free methods lead to very selective labeling patterns, but their use is often still hampered by high costs and limited applicability (Casteleijn et al. 2013).

The introduction of late α-ketoacid metabolic precursors for valine, isoleucine and leucine (Gardner and Kay 1998; Goto et al. 1999; Lichtenecker et al. 2004, 2013a, b), as well as methionine (Fischer et al. 2007) resulted in hitherto unrivaled labeling selectivity in cell-based protein overexpression. Further development led to techniques of stereoselective methyl labeling in leucine, valine or isoleucine (Ayala et al. 2012; Gans et al. 2010) and extended selective labeling to alanine and threonine (Ayala et al. 2009; Velyvis et al. 2012). The assembly of ^13^CHD_2_ methyl groups (Chaykovski et al. 2003; Ollerenshaw et al. 2005; Weininger et al. 2012b) provides optimized isotope patterns to probe for conformational changes. Compared to all these advanced techniques of aliphatic residue labeling (reviewed by Kerfah et al. 2015), the methods to introduce defined isotope patterns into aromatic residues extensively lagged behind for many years. This is all the more surprising, because phenylalanine, tyrosine, tryptophan and histidine are regarded as sensitive reporters of protein dynamics, as well as being valuable sources of structural restraints. In addition, these residues are significantly overrepresented at protein interfaces and play a prominent role in guiding enzyme mechanisms (Bogan and Thorn 1998). The absence of a comprehensive toolbox of amino acid precursors for selective aromatic residue isotope labeling inspired us to identify novel compounds, which show effective cell-uptake and well-defined in-vivo conversion to the target residues in *E. coli* overexpression systems.

## Precursor identification

Early metabolic precursors (biosynthetic intermediates upstream of the shikimate- or the pentose phosphate pathway) have been applied to achieve a certain isotope distribution in the corresponding target residues. Isotopologues of d-glucose (Teilum et al. 2006; Lundström et al. 2007, 2009a, b; Weininger et al. 2012a, 2013), glycerol (Ahlner et al. 2015; LeMaster and Kushlan 1996; Takeuchi et al. 2008), d-erythrose (Kasinath et al. 2013, 2015; Weininger 2017b), d-ribose (Weininger 2017a), acetate (Wand et al. 1995) and pyruvate (Guo et al. 2009; Lee et al. 1997; Lundström et al. 2008; Milbradt et al. 2015; Robson et al. 2018) have been particularly used to reduce unwanted ^1^J_CC_ coupling in NMR-based analysis of protein dynamics. These methods have been frequently applied by the biomolecular NMR community, since the corresponding precursors are commercially available and additional synthetic organic chemistry is not needed. However, a significant degree of cross-labeling cannot be avoided when using early metabolic precursors, which not only leads to heavy isotope incorporation at unwanted atomic positions, but also results in labeling of others than the target amino acids. Since the compounds mentioned above are central to the biosynthesis of all four proteinogenic aromatic residues, generating residue-selective isotope patterns is impossible. In addition, the poor selectivity causes limited maximal isotope incorporation levels, which is to some extent compensated by supplying increased precursor concentrations in the overexpression media. The resulting high consumption rate of isotope labeled compounds is a serious expense factor and limits the sample throughput of protein NMR spectroscopy.

**Scheme 1:**
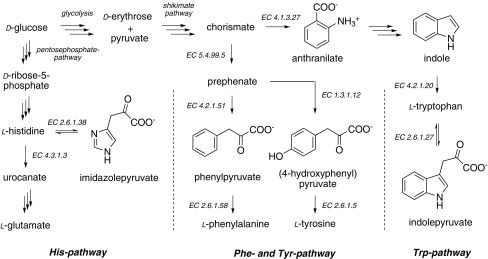
Outline of the aromatic amino acid metabolism in *E. coli*

In order to address the issues mentioned above, we identified late metabolic precursors for aromatic residue labeling. Phenylpyruvate and (4-hydroxyphenyl)pyruvate are the substrates of the transaminase catalyzed conversion to the corresponding target amino acids l-phenylalanine and l-tyrosine, respectively (Scheme 1) (Lichtenecker et al. 2013c). These two compounds represent the only non-chiral intermediates in the corresponding biosynthetic pathway and are thus ideal structurally simple targets for isotopologue synthesis (Lichtenecker 2014). The α-ketoacid derivative of tryptophan, indolepyruvate, is not part of the amino acid biosynthesis, but the first intermediate in the corresponding degradation pathway. However, we could identify this compound as a selective precursor for tryptophan labeling (Schörghuber et al. 2015). The reversible character of the corresponding transaminase *EC 2.6.1.27* reaction leads to efficient conversion of the precursor to the target residue in this case. In order to access isotopologues of the indole side-chain, we tested structurally more simple compounds for their use in selective tryptophan labeling. Considering the irreversibility of the anthranilate synthase *EC 4.1.3.27* catalyzed elimination of pyruvate from chorismate, we could provide evidence that isotope patterns in anthranilate, as well as indole can be transferred to the tryptophan side-chain without losing heavy isotopes in the shikimate pathway (Schörghuber et al. 2015, 2017a). Regarding histidine labeling, we focused on the first intermediate of the minor degradation pathway, imidazolepyruvate. Again, the reversible transaminase *EC 2.6.1.38* reaction ensured an effective in-vivo conversion to the target residue. In this case we applied the stable enol-tautomer of imidazolepyruvate as a labeling precursor (Schörghuber et al. 2017b). All of the identified precursor compounds mentioned showed highly selective labeling of the corresponding target residues in absence of any cross-labeling to undesired atomic positions.

## Precursor synthesis

**Scheme 2:**
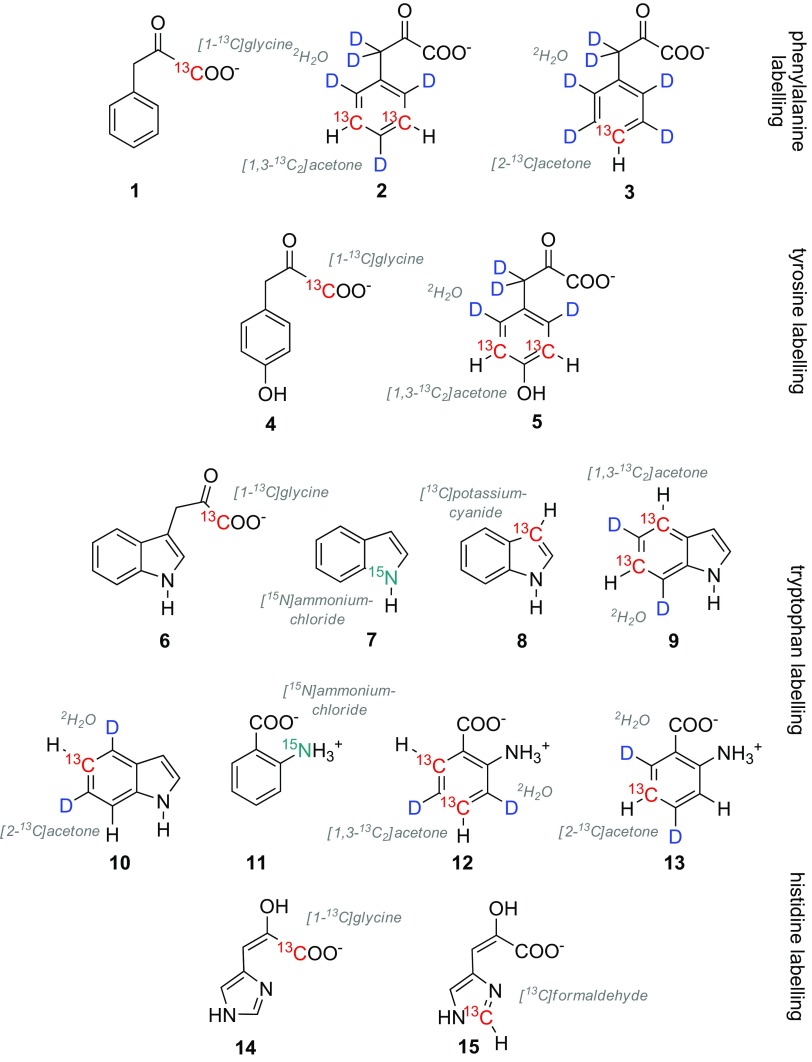
Late metabolic precursor compounds for aromatic residue labeling. Commercially available isotope sources are denoted in grey italics

Scheme 2 summarizes the precursors we used for aromatic residue labeling so far. These compounds have been prepared via multistep organic synthesis, which we optimized in terms of robustness, yields, labeling selectivity and costs (see the corresponding literature for details). We used commercially available sources of ^13^C as starting materials or reagents, such as isotopologues of acetone, glycine, potassium cyanide and formaldehyde (Lichtenecker 2014; Lichtenecker et al. 2015). Deuterium patterns have been exclusively derived from deuterium oxide, which is the cheapest source of ^2^H available. 1-^13^C labeled precursors **1, 4, 6** and **14** have been applied to probe for labeling selectivity and precursor uptake in diverse model protein overexpression systems. Their straightforward synthesis (**1, 4** and **6** are prepared from [1-^13^C]glycine in three steps) renders them economic tools for residue-selective backbone-labeling to be used for e.g. unambiguous signal assignment in high molecular-weight protein complexes.

**Scheme 3:**
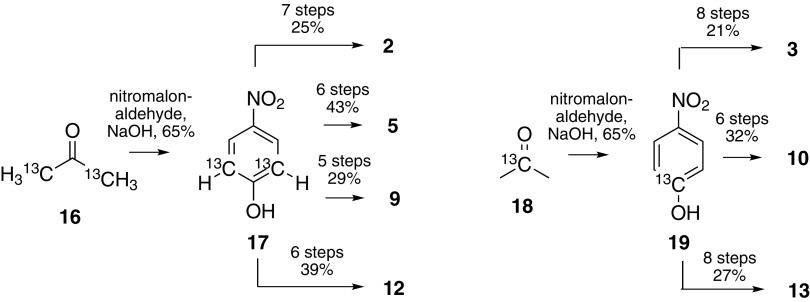
Synthesis of Phe-, Tyr- and Trp-precursors via the common synthetic intermediates [2,6-^13^C_2_]4-nitrophenol **16** and [1-^13^C]4-nitrophenol **19**. For more details concerning synthetic routes and concepts, see the corresponding literature (Lichtenecker 2014; Schörghuber et al. 2015, 2017a)

The Phe-precursors **2** and **3**, the Tyr-precursor **5**, as well as the Trp-precursors **9, 10, 12** and **13** display well-defined deuteration patterns leading to isolated ^13^C–^1^H spins. These systems are devoid of any additional scalar couplings, which otherwise distort relaxation rate analysis. Highly regioselective ^1^H/^2^H exchange was performed on electron-rich aromatic rings in acidic deuterium oxide under elevated reaction temperatures. All these compounds can be accessed from one common synthetic intermediate (isotopologues of 4-nitrophenol **17** and **19**), which is a significant advantage from an economic point of view (Scheme 3). Labeled histidines are important sensors for protein dynamics and help to elucidate the pK_a_ values of the imidazole ring (Hansen and Kay 2014; Hass et al. 2008). The ε-^13^C His-precursor **15** exhibits an inherently isolated ^13^C–^1^H spin system and was developed to provide an optimal isotope pattern to probe this unique heteroaromatic side-chain. Compound **15** can be prepared via a straightforward 5-step route starting from [^13^C]formaldehyde (Schörghuber et al. 2017b).

## Precursor uptake and labeling selectivity

Effective uptake of isotope labeled precursors by the overexpressing organism is of utmost importance, since this factor determines the highest possible isotope incorporation level at certain precursor concentrations in the corresponding media. Isotope incorporation at a given concentration may vary with different target proteins as a function of protein size, number of target residues and overexpression conditions. We identified the following precursor concentrations as being required in the *E.coli* overexpression media to achieve near-quantitative to quantitative isotope incorporation at the target atomic positions: 60–150 mg/L phenylpyruvate, 80–200 mg/L 4-hydroxyphenylpyruvate (Lichtenecker et al. 2013c), 20–60 mg/L indolepyruvate, 12–30 mg/L indole (Schörghuber et al. 2015), 8–30 mg/L anthranilic acid (Schörghuber et al. 2017a), and 50–100 mg/L imidazolepyruvate (Schörghuber et al. 2017b). This data has been deduced from labeling efficiency plots, which were obtained by overexpressing the corresponding protein sample in presence of different precursor concentrations. The low amount of late metabolic precursors required to achieve maximal heavy isotope contents is in sharp contrast to labeling methods using early metabolic intermediates. Examples from literature report concentrations of 1–4 g/L in this case, leading to isotope enrichment of 30–75% at the desired atomic positions in Phe, Tyr, Trp or His-residues (e.g. Kasinath et al. 2013; Weininger 2017b). These values are far from the quantitative isotope labeling, which we observed when applying the late metabolic precursor compounds illustrated in Scheme 2.

Using single atom ^13^C-labeled early metabolic precursors induces a certain pattern of ^12^C/^13^C isotopes, but despite of improving selectivity by exploiting auxotrophic organisms (e.g. LeMaster and Kushlan 1996) or supplying the growth media with enzyme inhibitors (e.g. Tong et al. 2008) a certain degree of cross-labeling cannot be ruled out. In various applications of our precursor toolbox, we did not observe any isotope scrambling so far. Our experiments indicate that any isotope pattern, which can be implemented onto the structures shown in Scheme 2 will quantitatively be transferred to the corresponding target residue. Most importantly, this is also true for patterns of (non-solvent exchangeable) deuterium atoms. Consequently, late metabolic precursors of aromatic residues can be applied to introduce aromatic ring protons into protein samples with high overall deuterium levels. In this case, uniform deuterium labeling can be achieved using deuterium oxide together with ^2^H-containing early metabolic isotope sources such as [*all*-^2^H]glucose.

Another strategy to achieve well-defined isotope patterns in protein samples applies labeled amino acids as additives to the overexpression media. Examples from literature show that amino acid concentrations in the low mg/L range may result in high incorporation levels (Kemple et al. 1994; Miyanoiri et al. 2011; Vuister et al. 1994). The concentrations required can even be further decreased when using auxotrophic expression strains (Lin et al. 2011; Yang et al. 2015). Other reports indicate that the use of labeled amino acids in *E. coli*-based overexpression systems is to some extent limited by metabolic product feedback control mechanisms, which may lead to decreased isotope uptake, retarded cell growth or cross-labeling (Krishnarjuna et al. 2011; O’Grady et al. 2012; Rowley 1953). In the case of aromatic residues, Phe, Tyr and Trp have shown to inhibit the *E. coli* DHAP synthase isoenzymes, which control the carbon flow into the shikimate pathway (Herrmann 1995). Additionally, certain levels of amino acid concentrations affect the translation machinery, thereby slowing down growth rates (Avcilar-Kucukgoze et al. 2016). Besides, organic synthesis of complex isotope patterns in the case of amino acids is considerably elaborate and expensive, due to the required implementation of at least one center of chirality, as well as the need of introducing ^15^N by additional synthetic steps if nitrogen-15 labeling is desired (Miyanoiri et al. 2011). In contrast to that, ^15^N-labeling of the target residues is straightforward in the case of applying metabolic amino acid precursors by adding ^15^N-salts to the expression media. In order to reduce synthetic efforts, more elaborate commercially available isotope sources can be applied. Phenylalanine, for instance, has been prepared from labeled tyrosine in two steps (Wang et al. 1999). The simplified synthesis, however, comes along with increased costs for the starting compound. In a recently published noteworthy economic approach, labeled phenylalanine was produced in *E. coli* from glycerol and secreted into the growth medium. The thus isolated amino acid subsequently served as an isotope source in recombinant *E. coli* protein overexpression (Ramaraju et al. 2017). However, the application of amino acids in cell-based overexpression required the addition of metabolic inhibitors and unlabeled amino acids in order to obtain high isotope incorporation levels also in this case. One literature reported protocol makes use of shikimic acid to generate protonated aromatic residues in an otherwise uniformly ^13^C-protein (Rajesh et al. 2003). It can be considered as rather improbable that this strategy will be transferred from reverse-labeling to selective ^13^C-labeling in aromatic residues in future, due to the required synthesis of ^13^C-shikimic acid.

Table 1 gives a -by no means exhaustive- overview concerning the various strategies of aromatic residue labeling in *E. coli* based and cell-free systems published so far. The data shown shall illustrate the differences in selectivity, precursor concentrations needed, synthetic effort and isotope incorporation levels.

**Table 1:**
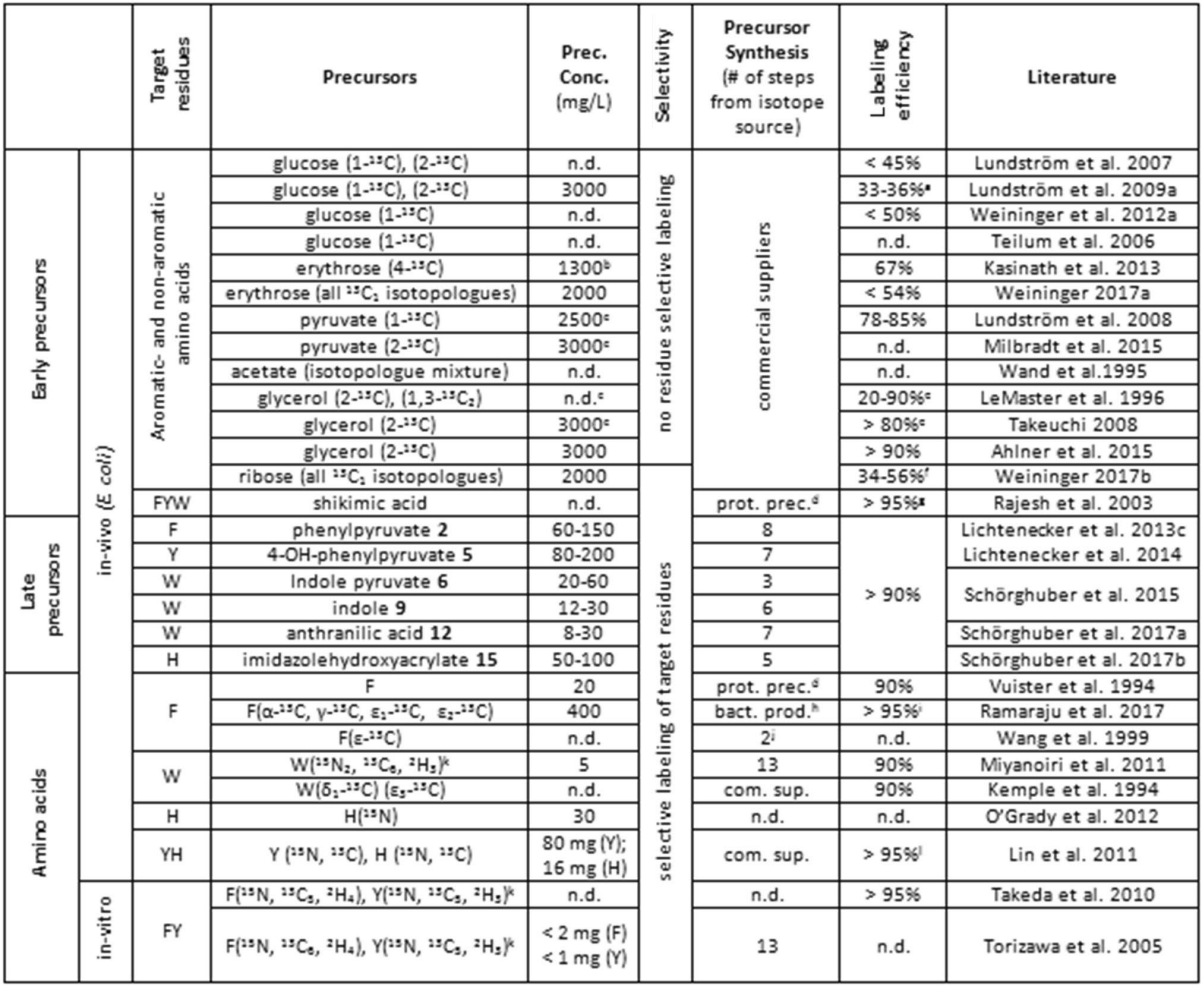
Overview of published protocols concerning aromatic residue protein labeling

*n.d*. no data available, *prec. conc*. precursor concentration, *prot. prec*. protonated precursor, *bact. prod*. bacterial production, *com. sup*. commercial suppliers, amino acids are depicted by one letter code

^a^Expression in a succinate dehydrogenase deficient *E. coli* strain

^b^Together with 2 g/L deuterated pyruvate

^c^Additional supply of NaH^13^CO_3_

^d^Reverse labeling using protonated precursor

^e^Overexpressing organism lacks succinate- and malate dehydrogenase; addition of NaH^13^CO_3_

^f^Incorporation rates can be maximized to 75% by addition of ^13^C-isotopologues of glucose

^g^Application of a shikimate auxotroph

^h^Precursor produced in *E. coli* medium containing [2-^13^C]glycerol

^i^Addition of glyphosate and unlabeled amino acids to prevent cross labeling and increase precursor uptake

^j^Synthesized from labeled tyrosine

^k^For the exact isotopic patterns of these precursors see the literature cited

^l^An auxotrophic overexpression host was used

## Applying late metabolic aromatic precursor compounds to investigate proteins and their interaction sites

### Chemical shift mapping

**Fig. 1:**
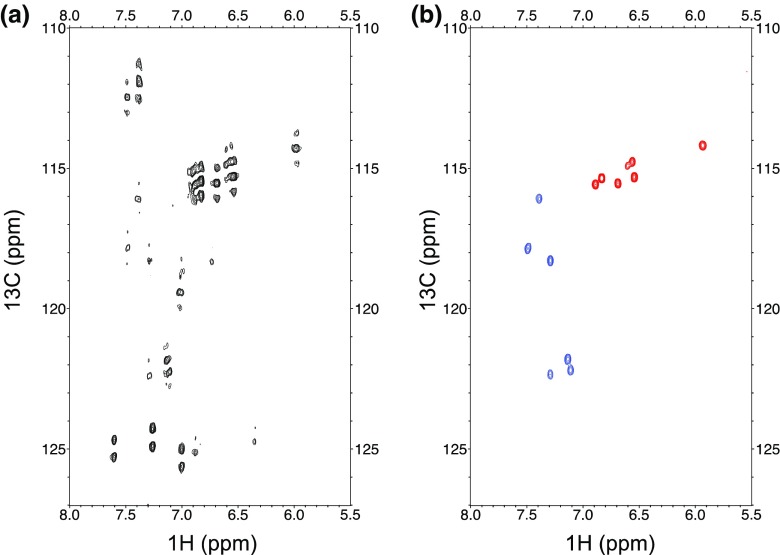
**a** ^1^H–^13^C HSQC spectrum of uniformly ^13^C-labeled Brd4-BD1. **b** ^1^H–^13^C HSQC spectra of selectively Trp(^13^C^ε3^/^13^C^η2^) (blue) and Tyr(^13^C^ε^) (red) labeled Brd4-BD1. Samples were expressed and purified as described before (Schörghuber et al. 2017a). Spectra were acquired on a 600 MHz spectrometer at 298 K on a sample of 0.1 mM protein concentration at pH 7.5

We chose bromodomain 1 of bromodomain-containing protein 4 (Brd4-BD1) as an example to highlight the benefits of labeling isolated positions in aromatic side-chains. Brd4 is a chromatin reader that binds to acetylated lysines in histones and has proven to be a promising cancer target in the pharmaceutical industry (Zeng and Zhou 2002; Sanchez et al. 2014). We already described Trp-labeled Brd4-BD1 in previous work (Schörghuber et al. 2017a), and we want to elaborate on the application of selective aromatic labeling for probing ligand interaction further using this system. Figure 1 compares the ^1^H–^13^C HSQC spectrum of uniformly ^13^C-labeled Brd4-BD1 (Fig. 1a) with the spectra of either Trp(^13^C^ε3^/^13^C^η2^)- or Tyr(^13^C^ε^)-labeled Brd4-BD1 (Fig. 1b, blue and red spectrum). Figure 1b shows six tryptophan- and seven tyrosine resonances according to the three ^13^C^ε3^/^13^C^η2^-labeled tryptophans, as well as the seven ^13^C^ε^-labeled tyrosines present in the Brd4-BD1 sequence. All the signals are well-defined and devoid of any splitting due to additional scalar couplings. On the contrary, the tryptophan resonances of the uniformly labeled sample barely exceed the noise threshold and all aromatic side-chain signals are affected by strong J_CC_ couplings to neighboring positions. Especially the Tyr-signals suffer from substantial signal overlap, which severely restricts their use for ligand induced chemical shift perturbation studies.

**Fig. 2:**
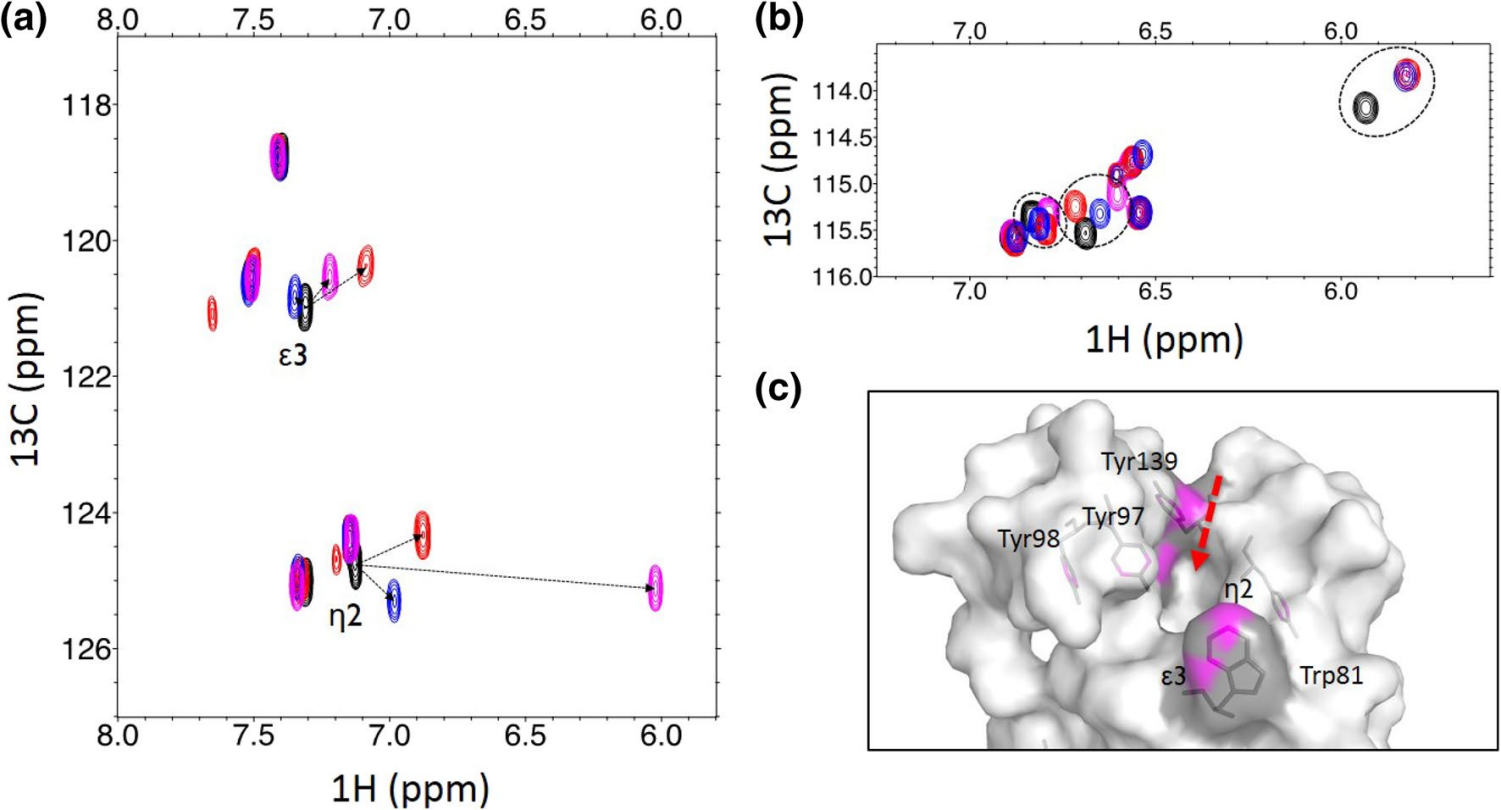
CSPs observed in ^1^H-^13^C HSQC spectra of selectively Trp(^13^C^ε3^/^13^C^η2^)-labeled Brd4-BD1 **(a)** and Tyr(^13^C^ε^)-labeled Brd4-BD1 **(b)** after the addition of three different ligands (red, blue and magenta spectra). The binding cleft of Brd4-BD1 is shown (red arrow) highlighting the proximal Tyrosine (Tyr97, Tyr98, Tyr139) and Tryptophan residues (Trp81) **(c)**

Figure 2 illustrates the chemical shift perturbations (CSPs), which have been induced by three different ligands in ^1^H–^13^C HSQC spectra of Trp(^13^C^ε3^/^13^C^η2^)-labeled Brd4-BD1 (Fig. 2a) and Tyr(^13^C^ε^)-labeled Brd4-BD1 (Fig. 2b). The corresponding protein samples were overexpressed in *E. coli* using precursor **12** (20 mg/L medium), or precursor **5** (100 mg/L medium) containing minimal medium, respectively. The low-molecular weight ligands added target the binding cleft of Brd4-BD1, which is lined by one tryptophan (Trp81) as well as two tyrosine residues (Tyr97 and Tyr139). The spectra reveal that the only resonances to be affected originate from residues in close contact to the binding site (Tyr97, Tyr139 and Trp81). The third shifting tyrosine resonance is very likely caused by Tyr98, which is in close contact to Tyr97 and thus also influenced by ligand binding. Importantly, strong CSPs in the proton dimension of selectively labeled sites provide crucial information about the proximity of aromatic ring-systems of interacting ligands. CSPs induced in the tryptophan spectrum (Fig. 2a) show only marginal differences between the three tested ligands. Only one of the compounds induces a strong upfield shift in the proton dimension of the η2-position of Trp81, indicating a binding mode, which places an aromatic ring-system on top of H^η2^ (Fig. 2a, magenta spectrum).

### Ligand induced cross-correlation rates

**Fig. 3:**
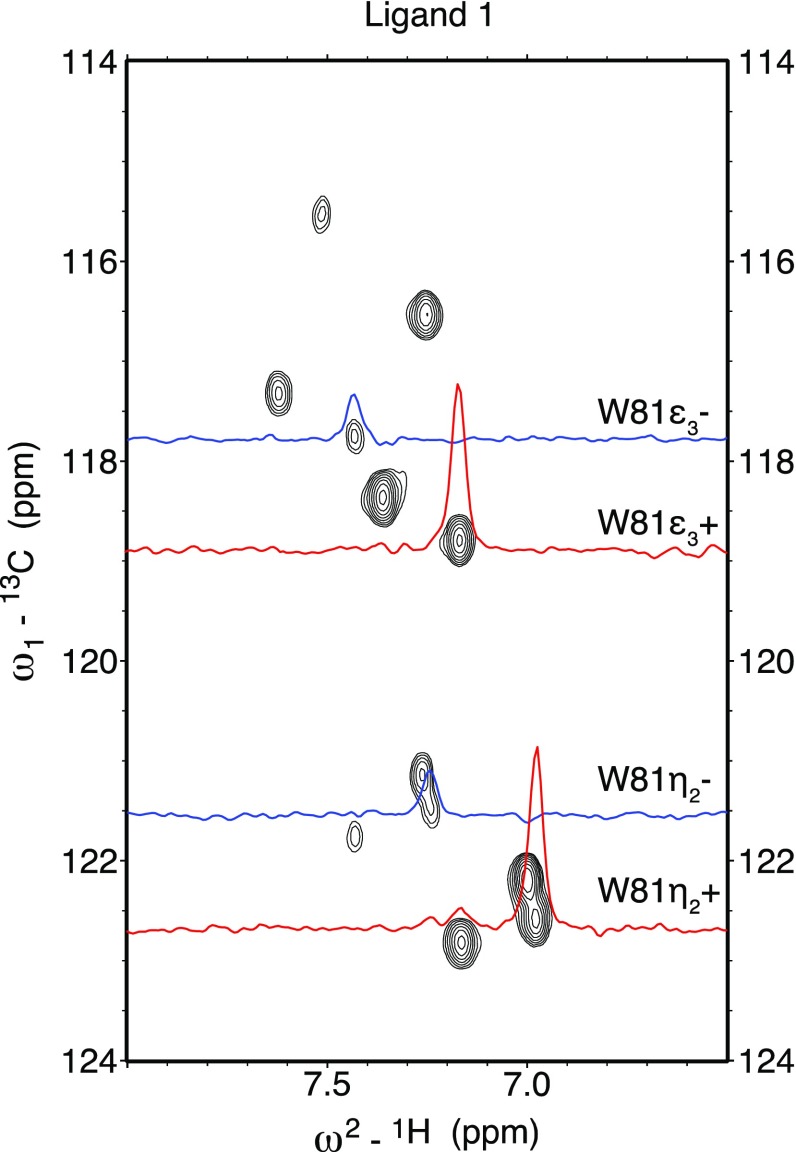
Coupled ^1^H–^13^C HSQC spectrum of selectively Trp(^13^C^ε3^/^13^C^η2^)-labeled Brd4-BD1 with 1D slices for Trp81 downfield (blue) and upfield (red) components. Samples were prepared and measured as denoted in Fig. 1

In additional experiments, we employed selective Trp ^13^C^ε3^/^13^C^η2^ labeling on Brd4-BD1 to evaluate the effect of different ligands on the Chemical Shift Anisotropy (CSA)–Dipol-Dipol (DD) cross correlation rate (CCR) η of the labeled carbon nuclei. As mentioned before, the Brd4-BD1 system comprises three tryptophans, one of which (Trp81) is embedded in a hydrophobic pocket that is targeted by various inhibitor molecules (Jung et al. 2014). Cross correlation between CSA and DD interactions in a system of coupled 1/2 spins can yield valuable information about local electronic structure and dynamics (Brutscher 2000; Kumar et al. 2000). Therefore, we chose precursor **12** to generate exclusive ^13^C^ε3^–^1^H/^13^C^η2^–^1^H spin pairs in the target residues. This pattern eliminates scalar coupling contributions from nearby C^ζ2^–H and C^ζ3^–H pairs. In order to calculate CCRs for Trp81^ε3^ and Trp81^η2^ we utilized a coupled version of a constant time ^1^H–^13^C HSQC to extract peak intensities for the upfield (I_+_) and downfield (I_−_) components of the ^13^C doublets, which allows for the calculation of corresponding cross-correlation rates. Figure 3 displays the twelve signals that emanate from the three tryptophan residues present. Highlighted are the 1D slices for both Trp81C^ε3^ and Trp81C^η2^ showing the upfield (+) components in red, and the downfield (−) components in blue. The ratio of peak intensities (I_+_/I_−_) is related to the CCR (η) via ln(*I*_+_/*I*_−_) = *2Tη* where T is the mixing time in the NMR experiment during which cross correlated relaxation is active (Brutscher 2000).

**Fig. 4:**
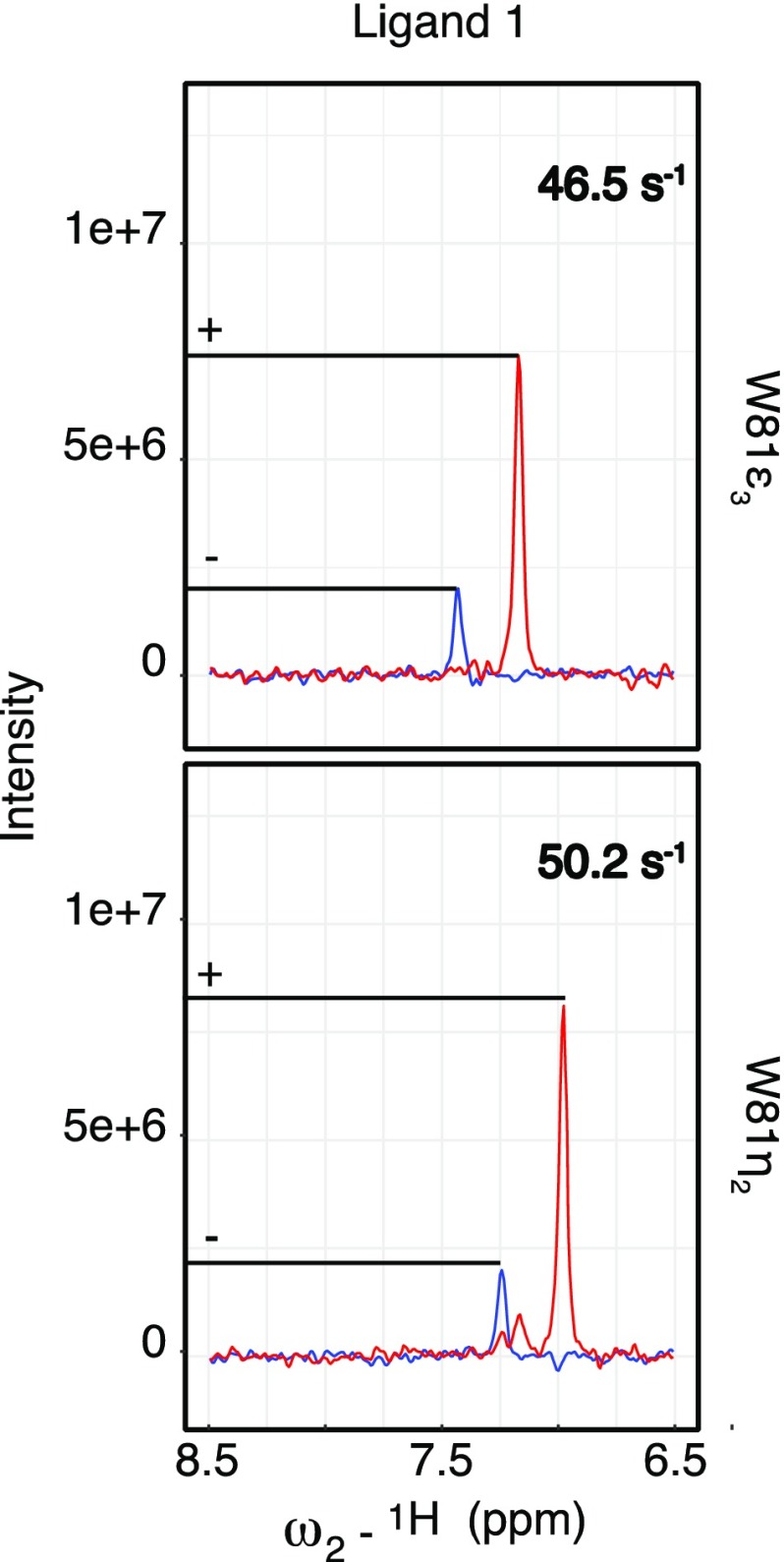
Overlay of the up- and downfield slices extracted for Trp81C^ε3^ (top) and Trp81C^η2^ (bottom) in the presence of Ligand 1 with their corresponding η values. Samples were prepared and measured as denoted in Fig. 1

Figure 4 shows 2D spectra and extracted 1D traces in the presence of a small molecule ligand and their corresponding η values. CCR rates for Trp81^ε3^ and Trp81^η2^ were determined to 46.5 and 50.2 s^−1^. Interestingly, these rates were smaller than in the apo-state of BRD4 (Trp81^ε3^: 50.8 s^−1^; Trp81^η2^: 53.3 s^−1^) presumably due to subtle changes of the CSA tensor and/or local conformational dynamics.

### Structural restraints in intrinsically disordered proteins

**Fig. 5:**
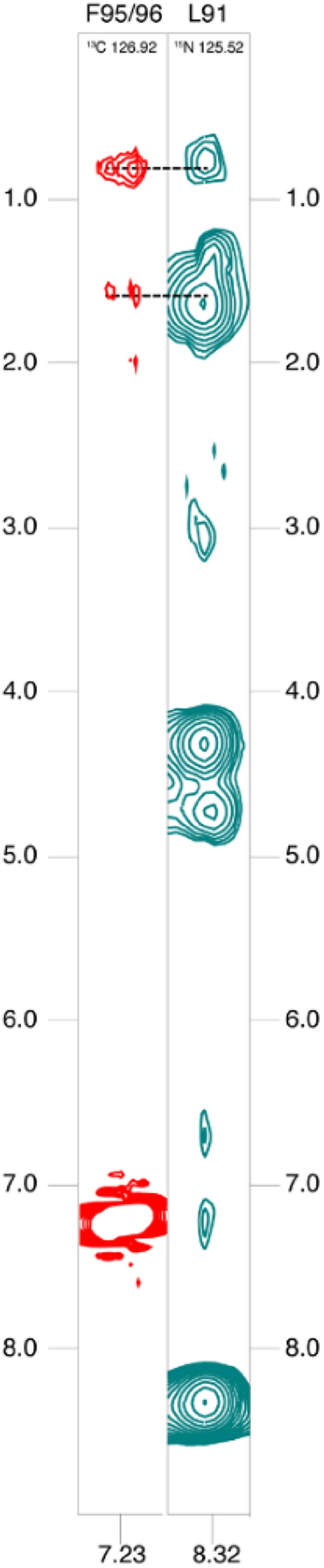
*Left* A strip from a ^13^C-NOESY-HSQC spectrum of the uniformly ^15^N and selectively phenylalanine labeled IDP YAP (50-171). The strip is assigned to Phe95/96-H^ς^ and exhibits NOEs to Leu91-H^γ^ and Leu91-H^δ^. *Right* A strip from a ^15^N-NOESY-HSQC of the same protein assigned to Leu91-H^N^. Spectra were aquired at 800 MHz, 298 K on a sample of 1.0 mM protein concentration at pH 6. ^13^C and ^15^N chemical shifts are indicated in the top region of the slices

Highly selective aromatic residue labeling holds promise to yield valuable additional distance information through well-defined NOE signals, even in the case of structurally flexible protein regions or intrinsically disordered proteins (IDPs). Various research groups are just beginning to explore the potential of IDPs as important targets in drug development (Zhang et al. 2015). The acquisition of structural restraints to identify potential preformation of specific secondary structure elements in the unbound state, as well as their conformational stabilization upon ligand binding, is however still a very challenging task (Konrat 2014). Figure 5 shows a ^13^C-NOESY-HSQC strip of an intrinsically disordered N-terminal fragment (residues 50-171) of Yes-associated protein (YAP). This region was identified to contain the binding site to the transcription factor TEAD (Vassilev et al. 2001). The YAP/TEAD interaction regulates the Hippo pathway, which is deregulated in various cancers and therefore represents a promising target for cancer therapy (Liu et al. 2012). A recent study indicates propensities for the preformation of an α-helix and an N-terminal β-strand in YAP50-171 in its unbound state (Feichtinger et al. 2018). These partially preformed secondary structural elements, together with an omega-loop (residues 86-100), form the interacting surface upon TEAD binding. For the omega-loop, no structural preformation was anticipated so far. In order to further investigate the potential preformation of certain structural elements in this IDP, a uniformly ^15^N-YAP 50-171 sample was additionally labeled using compound **3** in the corresponding growth medium. Significant long-range (side-chain) NOEs between Phe95/96-H^ς^ and Leu91-H^γ^ and Leu91-H^δ^ were observed (Fig. 5). This data shows that, although YAP is largely unfolded in absence of its binding partner, very selective labeling can identify direct distance proximities and thus long-range structural preformation of the Ω-loop region (residues 86-100). This property was not perceived by analyzing chemical shift data via secondary structure propensity score calculation (Marsh et al. 2006) in previous studies (Feichtinger et al. 2018).

## Conclusion

To conclude this article, we want to summarize the various reasons, which make us think that our ensemble of heavy isotope containing aromatic compounds can be considered as a valuable tool for the NMR-based investigation of structure and dynamics in different proteins, as well as the NMR-guided drug development process. The use of metabolic amino acid precursors downstream of the shikimic acid pathway ensures for highly selective labeling with maximum incorporation rates. The effective cellular uptake and direct in-vivo conversion to the target residues not only reduces the amount of labeled protein sample required, but also creates new opportunities to tune certain isotope patterns to the demands of the NMR experiments applied. Well-defined isotope distribution results in highly resolved sets of NMR resonances even in the case of high molecular weight protein targets, which can be probed for changes upon ligand addition in a straightforward way. Binding of different ligand structures can thus be compared without the need of laborious full signal assignment. Concerning our synthetic routes to generate special precursor isotopologues, we laid the focus on isolated spin systems, which detect dynamic properties without interfering additional ill-defined relaxation pathways. An aspect of special significance is given by the reduced content of our labeled compounds in the growth media required to achieve quantitative protein isotope incorporation levels. Our precursors can be synthesized in gram scale from very cheap isotope sources, which significantly helps to reduce costs in the otherwise very expensive sample preparation process.

We believe that aromatic residue labeling using late metabolic precursor compounds will further develop fields of biomolecular NMR, where highly selective labeling is an essential precondition for effective data acquisition. Examples include protein NMR-based drug development, in-cell NMR spectroscopy, studies of IDPs, the investigation of high-energy conformations, as well as the probing of very weak protein–protein interactions.
